# Cross cultural adaptation and psychometric properties of the Finnish version of Western Ontario shoulder instability index (WOSI)

**DOI:** 10.1186/s12891-022-06029-7

**Published:** 2022-12-24

**Authors:** Sami P Elamo, J P Kukkonen, T E Flinkkilä, J T Lehtinen, A K Joukainen, J J Paloneva, K K Lehtimäki, T T Kauko, V O Äärimaa

**Affiliations:** 1 Department of Orthopedics and Traumatology, Satakunta Central Hospital, Sairaalantie 3, 28500 Pori, Finland; 2 Turku University Hospital and University of Turku, Turku, Finland; 3 Oulu University Hospital, Oulu, Finland; 4 Tampere University Hospital (Hatanpää), Tampere, Finland; 5 Kuopio University Hospital, Kuopio, Finland; 6 Central Finland Healthcare District, University of Eastern Finland, Jyväskylä, Kuopio, Finland; 7 Auria Clinical Informatics, Hospital District of Southwest Finland, Turku, Finland

**Keywords:** WOSI, Western Ontario shoulder instability index, Shoulder, Instability, PROM, Patient reported outcome measures

## Abstract

**Background:**

Western Ontario shoulder instability index (WOSI) is a widely used disease-specific self-assessment measurement tool for patients with shoulder instability. The main aim of this study was to translate and cross culturally adapt the WOSI into Finnish language and to test its measurement properties.

**Methods:**

WOSI was translated in Finnish and adapted into an electronic user interface. 62 male patients with traumatic anteroinferior shoulder instability, programmed for stabilizing shoulder surgery, answered the questionnaire twice preoperatively (2 and 0 weeks), and twice postoperatively (3 and 12 months). Additional scoring tools, such as satisfaction to treatment outcome, subjective shoulder value (SSV), Oxford shoulder instability index (OSIS) and Constant score (CS), were used as comparators. The reliability, validity and responsiveness of WOSI were investigated through statistical analysis.

**Results:**

Preoperative test-retest results were available for 49 patients, and 54 patients were available at final follow up. The mean WOSI was 57.8 (SD 20.3), 70.4 (SD 18.9), and 85.9 (SD 15.5), at baseline, 3, and 12 months, respectively. There was a statistically significant mean improvement of 28.8 (SD 24.5) in WOSI between baseline and 12 months (p < 0.0001). The intraclass correlation coefficient for the preoperative WOSI was excellent 0.91. At 12 months WOSI had an excellent Pearson’s correlation coefficient both with SSV (0.69), OSIS (-0.81), and poor with CS (0.25) scores, confirming our a priori hypothesis. There were no detected floor nor ceiling effects for WOSI pre- or postoperatively. The calculated minimal detectable change was 9.2 and the estimated minimal clinically important difference 13.4 to 18.1.

**Conclusion:**

Finnish version of WOSI is a reliable and valid tool for assessing health state and improvement after operative treatment of shoulder instability in young male patients.

## Introduction

Shoulder dislocation and subsequent instability is often a trauma related condition involving especially young men [1, 2]. Western Ontario Shoulder Instability Index (WOSI) was developed by Alexandra Kirkley [3] as a patient reported outcome tool for evaluating disease specific quality of life in patients with shoulder instability. This scoring method WOSI was designed to capture all aspects of health i.e. mental, physical and social, as defined by the World Health Organization [4]. WOSI score includes 21 items in 4 question domains, i.e. physical symptoms and pain, recreation and work, lifestyle and social functioning, and emotional well-being. The total score can be expressed as an index, i.e. percentage of the best possible health state [3]. WOSI has been evaluated by the collaborative working group for health outcome measures (consensus based standards for the selection of health measurement instruments, COSMIN) as the most reliable and valid available tool for assessing patient reported outcome in treatment of shoulder instability [5–7].

In order to capture the patient perception of shoulder instability, the outcome should be measured in patient’s native tongue. Furthermore, potential cultural differences with regard to question formulation should be considered [8]. These linguistic and cultural differences can potentially jeopardize e.g. the comparison of results unless proper cross-cultural validation and evaluation of the psychometric properties are not performed [9]. Hitherto, there has not been a Finnish version of WOSI nor reports on its’ performance in a Finnish patient population.

The purpose of this study was to cross culturally adapt and evaluate the performance and psychometric properties, regarding reliability, validity and responsiveness, of the Finnish version of WOSI in a Finnish speaking cohort of patients with traumatic anteroinferior shoulder instability.

## Methods

The study was conducted as part of FINNISH trial (Clinicaltrial no NCT01998048, 28/11/2013). Approval for conducting this study was obtained from the Hospital District of Southwest Finland (T145/2013, decision TO1/004/13, 16/09/2013). Written informed consent was obtained from all the patients willing to participate the study. We conducted the study according to the revised Declaration of Helsinki by The World Medical Association [10].

### Translation process

A two-way translation of the original WOSI score was carried out as described by Beaton et al. [11]. Two native Finnish speaking shoulder surgeons and one professional Finnish translator translated the original English questionnaire to Finnish (stage I, forward translation). Then synthesis was made and possible discrepancies were resolved (stage II, synthesis). Equality of sense, rather than vocabulary, was given priority. Thereafter two native English speaking translators (one with specific expertise in medical terminology) back-translated the questionnaire to English (stage III, back translation). A consensus meeting with all five translators, a scoring methodologist, and a senior expert member was held, and the final translation was agreed on (stage IV, expert committee review). The whole process and all decisions were documented. Minor changes were made during the adaptation process to improve cultural relevance.

### Pre-testing

The translated Finnish WOSI was adapted into an electronic user interface and pre-tested (stage V, pre-testing) by the first author (SE) for comprehensibility with 10 healthy medical and non-medical personnel volunteers at Turku University hospital.

### Study population

Sixty two male patients undergoing stabilizing surgery for anterior shoulder instability were recruited at eight institutions in Finland during 2013–2017. The inclusion criteria for this trial were as follows: male patient under 25 years of age, clinically documented instability after traumatic shoulder dislocation and patient’s willingness for operative treatment. Exclusion criteria were as follows: non-congruency of the joint on imaging investigations, concomitant injuries affecting the shoulder, previous surgery of the ipsilateral shoulder, intellectual disability or disability to co-operate, patient’s denial. Patients were requested to answer the electronic WOSI twice, with a two-week interval just before elective surgery.

The patients were operated on by experienced shoulder surgeons with an arthroscopic Bankart method. Postoperatively the arm was immobilized in a simple sling for three weeks and thereafter physiotherapy training was commenced. The electronic WOSI was repeated at three months and one year postoperatively in addition to Subjective shoulder value (SSV) [12], Oxford shoulder instability score (OSIS) [13] and Constant score (CS) [14].

**Fig. 1:**
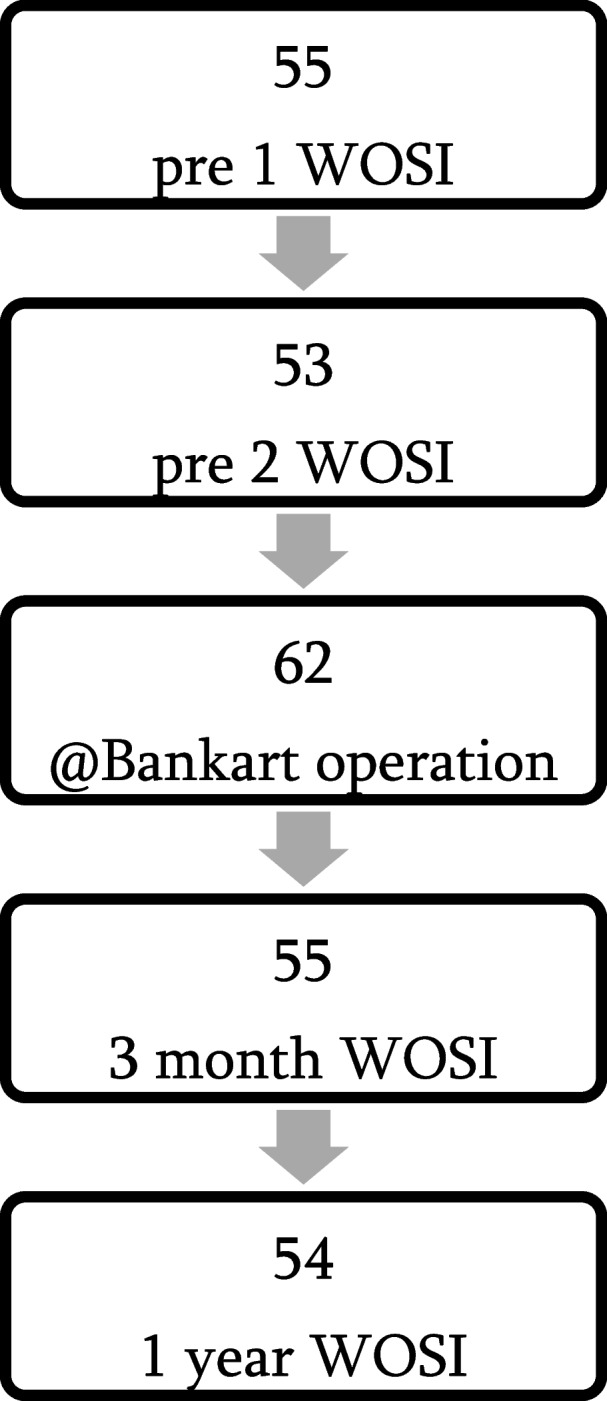
Flow chart. Number of patients available at certain time points of this study (pre 1 WOSI = Western Ontario Shoulder Instability (WOSI) questionnaire in baseline, pre 2 WOSI = WOSI in 2 weeks interval right before the surgery, @Bankart = arthroscopic Bankart operation, 3 month WOSI = WOSI questionnaire in 3 months post op, 1 year WOSI = WOSI questionnaire in 1 year post op

Subjective shoulder value (SSV) is a score where patient’s subjective shoulder assessment is expressed as a percentage of a totally normal shoulder (that would be 100%) [12]. Oxford shoulder instability score (OSIS) is a 12-item questionnaire where the questions cover different areas of life such as shoulder dislocations, trouble with dressing, worst or usual level of pain, avoidance of activities or prevented activities of importance, interference with work, social life, hobbies or lifting, avoided positions in bed at night or simply shoulder on your mind, adding up to 48 points in total representing the worst clinical result [13]. Constant score (CS) is a 100-point score for assessing the shoulder condition. Questionnaire’s 4 parameters are pain (15), activities of daily living (20), range of motion (40) and power (25) adding up 100 points in total representing the best clinical result [14]. An additional dichotomous anchor question: is your shoulder better or worse after surgery, was asked at three months. A flow chart for this study is presented in Fig. 1.

### Statistical analysis

#### Reliability

The internal consistency of WOSI and subdomains was calculated using Cronbach’s Alpha ((p / (p – 1)) (1 – (∑σ_i_^2^ / σ_X_^2^)), where p = number of items in WOSI (here, p = 21), σ_i_^2^ = variance of the *i*th item, and σ_X_^2^ = variance of the observed WOSI total score), with a value above 0.8 regarded as excellent [15]. The reliability was tested comparing the test-retest results through Bland-Altman method and calculating the intraclass correlation coefficient (ICC) for the total score, and the four domains. ICC above 0.75 was regarded as excellent, and below 0.4 as poor [15]. The measurement error (standard error of measurement, SEM) was estimated by calculating minimal detectable change (MDC) with the following equations: SEM = SD ×  √ (1 – ICC(3,k)), where ICC(3,k) is equal to Cronbach’s Alpha [16] and MDC = SEM x 1.96 x √ 2, where 1.96 is derived from the 95% confidence interval and √2 from 2 measurements at preoperative timepoint [17].

#### Validity

The construct validity was assessed through calculating Pearson’s correlation coefficient for SSV, OSIS and CS. We hypothesized a high positive correlation between WOSI domains and SSV, and a negative correlation between WOSI domains and OSIS. A correlation above 0.6 was regarded excellent and correlation below 0.3 poor [15]. 80% agreement was anticipated to achieve sufficient construct validity.

#### Responsiveness

The clinical outcome data was analyzed using primarily the analysis of variance of repeated measurements preoperatively, at three months and one year postoperatively. The proportion of responses hitting the lowest or highest limit of WOSI exceeding 15% was regarded as a significant floor and ceiling effect [18]. Both effect size (mean change divided by baseline standard deviation, ES) and standardized response mean (mean change divided by standard deviation of the change, SRM) were calculated, and we anticipated a large effect size. The ability of WOSI to detect change after the intervention (internal responsiveness), was determined by pooled ES and SRM. In addition, the ability of WOSI to detect a clinically important change with reference to an external anchor (external responsiveness) was determined by area under the receiver-operating characteristic (ROC) curve using the trapezoidal method [19]. The triggering change was determined by maximizing Youden’s J (sensitivity + specificity – 1). We used the dichotomous anchor question at three months to calculate the minimal clinically important difference (MCID). The triggering change in the WOSI associated with the response in the anchor question was determined by analysis of (1) the mean change, and (2) receiver-operating characteristic (ROC) analysis [20].

All analyses were performed using R version 3.6.3 (R Foundation for Statistical Computing, Vienna, Austria).

## Results

Fifty four patients were available at final follow up (drop-out rate 13%). Patient demographics are presented in Table 1. Complete test-retest results were available for 49 patients with a mean time of 1.0 (SD 1.4) and 11.6 (SD 4.5) days before surgery. There were no operative complications. 52 (96.3%) patients reported their shoulder to be better at three months compared to the preoperative state.

**Table 1:** Demographics

	Bankart operated (*n* = 62)	Available at final follow-up (*n* = 54)	Complete test-retest results available (*n* = 49)
Side (right/left)	21/41	19/35	17/32
Mean age at primary dislocation, years (SD)	18.6 (2.8)	18.5 (2.8)	18.6 (2.8)
Mean number of dislocations before surgery	13	13	13
Mean age at surgery, years (SD)	21.4 (2.7)	21.4 (2.7)	21.4 (2.8)
Weight, kg (SD)	76.5 (16.8)	76.3 (16.9)	76.5 (17.8)
Height, cm (SD)	178.5 (6.4)	178.3 (6.4)	178.1 (6.6)

*SD* standard deviation

### Reliability

**Fig. 2:**
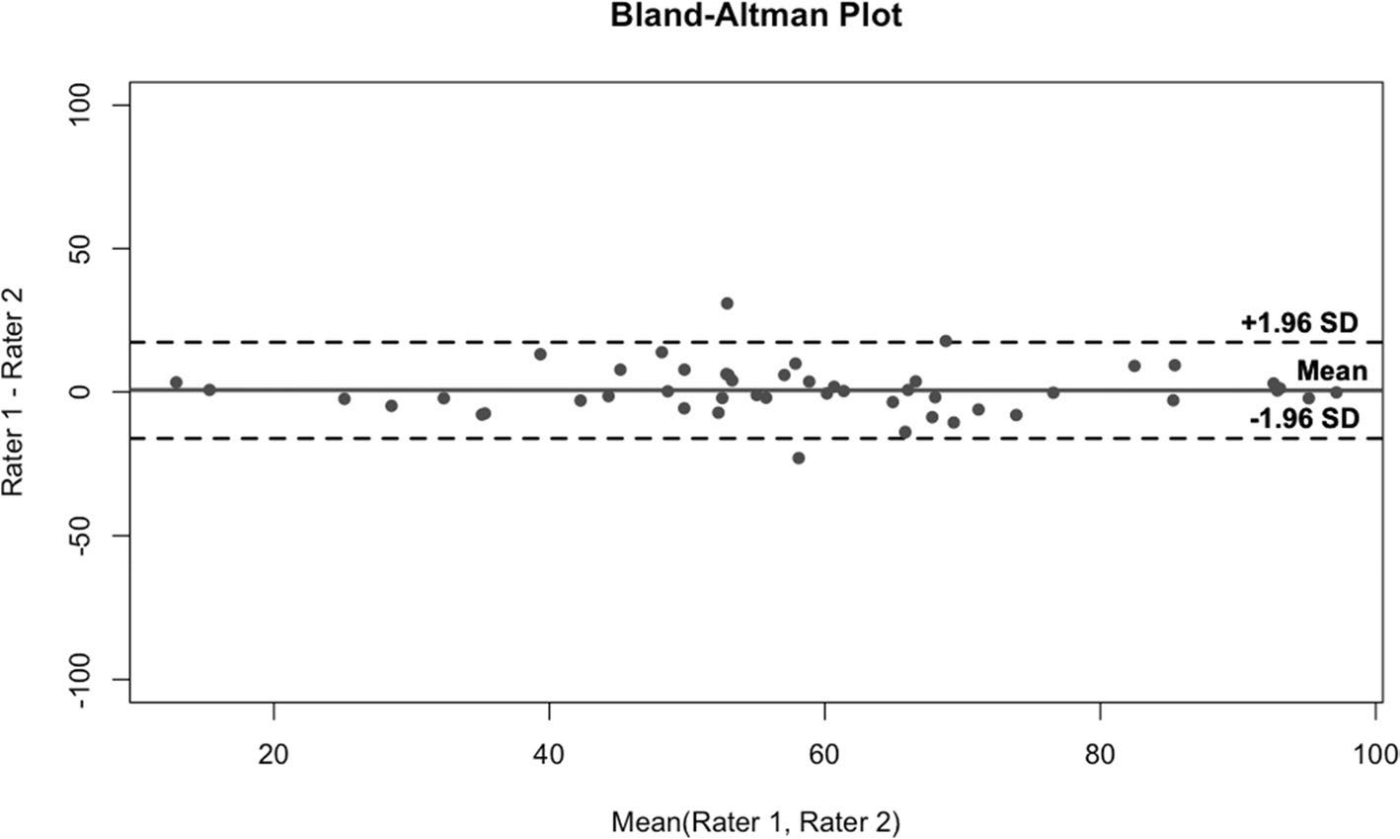
Agreement between WOSI test-retest measurements

The internal consistency, Cronbach’s alpha, was excellent 0.95 for the total WOSI, and 0.93, 0.81, 0.74, and 0.82 for the domains of physical symptoms and pain, recreation and work, lifestyle and social functioning, and emotional well-being, respectively. The test-retest ICC for the whole preoperative score was similarly excellent 0.91. The ICC for domains of physical symptoms and pain, recreation and work, lifestyle and social functioning, and emotional well-being were 0.89, 0.87, 0.90, and 0.81, respectively (Table 2). The test-retest Bland-Altman graph is presented in Fig. 2. The calculated MDC was 9.2 (SEM 3.31) for the total WOSI.

**Table 2:** Pearson’s correlation coefficient (r), intraclass correlation coefficient (ICC), and floor and ceiling effect of the four WOSI domains (physical symptoms and pain, recreation and work, lifestyle and social functioning, and emotional well-being) and total WOSI (95% confidence interval)

**WOSI domains**	**r**			**ICC**	**Floor/ceiling effect**	
	**SSV**	**OSIS**	**CS**		**Baseline**	**1 year**
Physical/pain	0.58 (0.37; 0.74)	-0.67 (-0.48; -0.80)	0.21 (-0.07; 0.46)	0.89 (0.83-0.93)	0 / 0	0 / 2%
Recreation/work	0.71 (0.54; 0.82)	-0.81 (-0.69; -0.89)	0.25 (-0.03; 0.49)	0.87 (0.80-0.91)	0 / 0	0 / 6%
Lifestyle/social	0.64 (0.45; 0.78)	-0.79 (-0.65; -0.87)	0.21 (-0.07; 0.46)	0.90 (0.85-0.93)	0 / 2%	0 / 6%
Emotional	0.68 (0.51; 0.81)	-0.79 (-0.66; -0.88)	0.26 (-0.02; 0.50)	0.81 (0.73-0.87)	3% / 0	0 / 8%
**WOSI total**	**0.69 **(0.52; 0.81)	**-0.81 (-0.69; -0.89)**	**0.25 **(-0.03; 0.49)	**0.91 (0.86-0.95)**	**0 / 0**	**0 / 0**

*WOSI* Western Ontario Shoulder Instability Index, *SSV* Subjective Shoulder Value, *OSIS* Oxford Shoulder Instability Score, *CS* Constant Score

### Construct validity

Postoperatively at one year WOSI had an expected and excellent Pearson’s correlation coefficient both with SSV (0.69), OSIS (-0.81), and poor correlation coefficient with CS (0.25) scores, confirming our a priori hypothesis. Table 2.

### Responsiveness

**Fig. 3:**
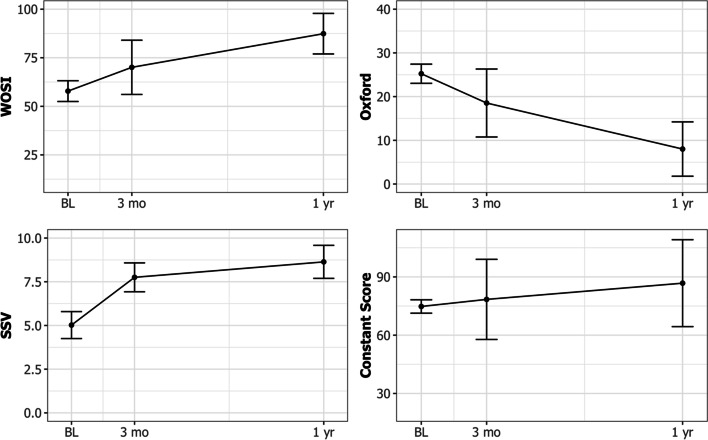
Graphical presentation of mean WOSI, SSV, OSIS and CS outcome scores with 95% confidence intervals (whiskers) at baseline, 3 months and 1 year post-operatively

The mean WOSI was 57.8 (SD 20.3), 70.4 (SD 18.9), and 85.9 (SD 15.5), at baseline, three months and one year, respectively. There was a statistically significant mean improvement of 28.8 (SD 24.5) in WOSI between baseline and one year follow up (p < 0.05). The 12-month effect size was 1.42 and standardized response mean 1.18. Graphs of all the outcome scores are presented in Fig. 3.

**Fig. 4:**
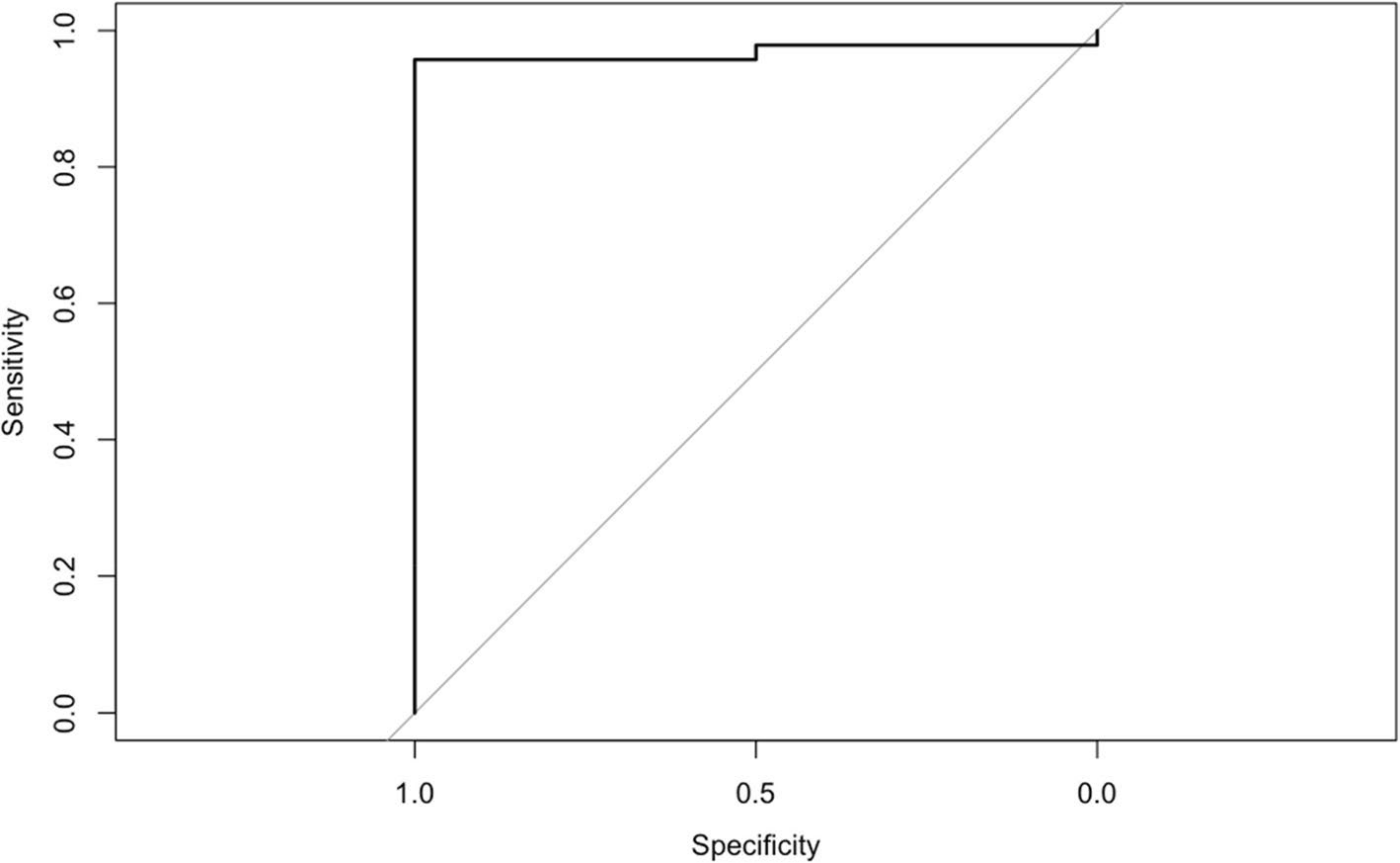
ROC sensitivity and specificity analysis of the change in the WOSI. ROC = receiver-operating characteristic, WOSI = Western Ontario Shoulder Instability Index

There were minor non-significant floor and ceiling effects in the subdomains, but not it the total WOSI pre- nor postoperatively. The calculated MCID for WOSI was 13.4 and 18.1 on the mean change and the ROC methods respectively. Area-under-curve (AUC) for the ROC curve was 0.97 (DeLong 95% CI 0.92-1.00) with 0.96 sensitivity and 1.00 specificity Fig. 4.

## Discussion

The Finnish version of WOSI was found to be both reliable and valid for this patient cohort as hypothesized. All test results of the questionnaire with regard to reliability, validity, responsiveness can be regarded as excellent. During the translation process some differences in interpretation were found within the working group. For example WOSI question number 17 (“How much difficulty do you have “roughhousing” or “horsing around” with family or friends”) brought up two different explanations. Where others believed it was about “domestic violence” others believed it to be “intimate moment with significant other”. The final Finnish translation consensus is more about “play fight with kids”. These findings exemplify the importance of careful contemplation while designing questionnaires to ensure proper item validity. Interestingly Drerup et al. [21] payed attention to the same question in their German translation process, pointing out the need for cross-cultural adaptation before administering patient reported outcome measurement tools.

WOSI has been previously translated, validated and culturally adapted at least in Swedish [22], German [21], Japanese [23], Italian [24], Dutch [25], French [26], Turkish [27], Danish [28], Hebrew [29], Polish [30] and Portuguese [31]. Reported intraclass correlation coefficient (0.87 to 0.99) and Crohnbach alpha (0.84 to 0.97) in these studies were comparable to the results in our study. With regard to construct validity we detected a high agreement with WOSI and also commonly utilized SSV and OSIS outcome scores for shoulder instability. A similarly sufficient construct validity for WOSI has been previously reported [25].

The test-retest interval in this study was short. We wanted to recapture the health state of patients using the shortest time interval possible so that patients would no longer accurately remember their previous responses. The method of re-testing during 1–4 week time-interval has been previously recommended by Beaton et al. [11] and Scholtes et al. [32]. Longer time interval could have altered the health state, and on the other hand shorter time could have facilitated a potential carryover effect with similar answers through memory. The repeated ratings in our study were similar, there were no indication of disagreement at any point on the scale, and the individual points scattered mostly within the desired standard deviation.

Structural recording of patient health states both before and after the intervention allows quantification of the change in the two health states i.e. subsequent treatment effect, and therefore assessment of the success of administered intervention [33]. Our results corroborate previous findings of inadequacy of Constant score when evaluating shoulder instability, and the need for a more specific and sensitive outcome measure [34]. In our study there was a marked improvement in patient’s health state after the intervention in terms of WOSI. Although this change was well above the measured MCID values, the benefit of arthroscopic Bankart operation is inconclusive based on findings in this study without a placebo treatment comparator.

The estimated MCID of 13% and 18% presented in this paper are rough mathematical estimates and may be different in other patient populations, and therefore should be considered highly theoretical. In order to catch potential non-recuperated patients, the 3-month change in WOSI was used for calculating the MCID [20]. Nevertheless, it is noteworthy that the number of non-satisfied patients was very low, and a big proportion of patients reporting high improvement in a rapidly recovering population plausibly increased the computational estimate for MCID especially with in this case skewed ROC calculation. Originally Kirkley reported a MCID of 10% for the WOSI, and a limit of moderate improvement to be 22% [35]. Our results are in accordance with these findings, and it is also noteworthy that our calculated measurement error for WOSI was below the estimated MCID value.

The prospective trial setup with a homogenous patient population, and a relatively low drop-out rate are strengths of this study. Symptomatic anteroinferior instability is a frequent problem after a traumatic shoulder dislocation in young males [36]. Young males are also reportedly challenging to treat [37], and therefore we specifically wanted to focus on this group of patients. On the other hand, as a clear limitation, patients with female gender, atraumatic onset of symptoms, and older age were excluded and may behave differently in their reporting although the previously reported psychometric estimates of WOSI, in more heterogenous patient cohorts, are essentially similar. We neither included patients with conservative treatment only. Therefore, the mathematical values presented in this study may not be applicable to all Finnish patients with shoulder instability. The evaluation of validity in our study may be regarded as constricted, as we have not performed an analysis of structural validity with methods of factor analysis. It is also noteworthy that the comparators for WOSI in our study are not scientifically cross culturally adapted nor validated in Finnish. Furthermore, the anchor question used to calculate the MCID was dichotomous and did not allow the patients to report a stationary health state in our study. This may have also influenced the presented MCID estimate, however no general consensus exists over the construct of an optimal anchor question when evaluating MCID.

## Conclusion

The Finnish version of WOSI is a reliable and valid tool in the assessment of health state and improvement after operative treatment of shoulder instability in young male patients.

## Data Availability

The datasets used and/or analysed during the current study are available from the corresponding author on reasonable request.
